# Resolution of Dizziness Following Treatment for Chronic Rhinosinusitis: A Case Report From Physical Therapy Outpatient Setting

**DOI:** 10.1155/crot/6175181

**Published:** 2025-11-08

**Authors:** Shellie Zsoldos, Chia-Cheng Lin

**Affiliations:** Department of Physical Therapy, East Carolina University, Greenville, North Carolina, USA

**Keywords:** case report, dizziness, sinusitis, vestibular rehabilitation

## Abstract

**Background and Purpose:**

The purpose of this case report is to describe dizziness symptoms caused by sinusitis evaluated in outpatient physical therapy setting.

**Case Description:**

A 59-year-old man with chronic peripheral vertigo of the right ear was referred to our outpatient physical therapy for vestibular rehabilitation. He was diagnosed with chronic peripheral vertigo of the right ear. He experienced a spinning sensation lasting for several hours, with no clear or consistent trigger for the onset of his dizziness.

**Intervention:**

Vestibular-ocular reflex (VOR) exercises were prescribed as part of a home exercise program.

**Outcomes:**

The patient was unable to perform the prescribed VOR exercises due to his travel schedule. However, he reported complete resolution of dizziness symptoms following surgical intervention and pharmacological treatment for chronic rhinosinusitis.

**Discussion:**

This case highlights the importance of a holistic approach when evaluating and treating patients with vestibular symptoms. Clinicians should consider sinus-related conditions, such as chronic rhinosinusitis, as potential contributors to dizziness.

**Summary**

• In this patient, the resolution of dizziness and aural symptoms following sinus surgeries emphasizes the importance of treating underlying chronic sinusitis in the management of dizziness symptoms.
• A holistic approach to evaluating and treating patients with vestibular symptoms should include thorough assessment of contributory sinus symptoms, careful review of medications, and tailored physical therapy interventions.
• With an interdisciplinary approach of ENT specialists and surgeons, physical therapists can enhance patient outcomes and address all contributory factors to vestibular dysfunction by integrating sinus health into a vestibular evaluation and treatment plan.

## 1. Introduction

Rhinosinusitis (RS) is caused by inflammation of the mucosal lining in the sinus cavities and the nasal fossae. The 2021 International Consensus Statement on Allergy and Rhinology defined that the diagnosis of RS should include nasal blockage/obstruction/congestion or discolored nasal discharge and facial pain or reduction/loss of smell [1]. If the symptoms last up to 12 weeks, it is classified as chronic rhinosinusitis (CRS) [1].

CRS-induced dizziness was first reported by Watson-Willams in 1924 in a person with sphenoidal and ethmoidal sinusitis [2]. Since that, a total of five case reports were found [3–7]. Dietz de Loos reported an incidence of 29.6% in people with CRS having dizziness, but the symptom scores were mild based on Sino-Nasal Outcome Test (SNOT-20) [8]. Although more research is needed to investigate the mechanism of CRS-induced dizziness, a few studies suggest that abnormal vestibular examination findings may present in people with CRS [9–12]. However, the abnormal vestibular examination findings may resolve after the treatment for RS [7, 9].

During vestibular assessment in physical therapy, CRS may not be considered a potential factor that contributes to a patient's dizziness due to the lack of knowledge and clinical evidence in CRS-induced dizziness. The present case report illustrates the importance of screening other systems/diseases that cause dizziness and clinical reasoning during vestibular assessment in outpatient physical therapy settings.

## 2. Case Description

### 2.1. Patient History

This case report was reviewed by the University and Medical Center Institutional Review Board (UMCIRB) at East Carolina University. It was determined not to meet the definition of human subject research based on the IRB's case report criteria.

A 59-year-old male was referred to our outpatient physical therapy clinic by his primary care physician for chronic peripheral vertigo of the right ear. He presented with reports of dizziness and a spinning sensation lasting for hours, without identifiable triggers or association with postural changes. Acute exacerbations occasionally occur after sleep, engaging in house projects, or driving; however, he denied consistent reproduction of symptoms. He also described cavitations in the upper cervical spine, occasionally followed by nasal drainage. Notably, he reported decreased dizziness after a good night's sleep.

The patient's dizziness symptoms were also accompanied by aural fullness, pressure, and ringing in the right ear, followed by nausea and vomiting depending on the severity of the symptoms. The patient reports that these symptoms began after contracting SARS-CoV-2 (COVID-19) 2 years ago. He is a retired Air Force pilot with a known history of right-sided nonpulsatile tinnitus and hearing loss. Following the viral infection, the patient began experiencing what he describes as an “unbearable heartbeat in the ear,” which is worsened by sinus congestion and often coincides with vertigo attacks.

His medical history includes psoriasis, hypertension, and seasonal allergies, for which he takes Zyrtec and Flonase during peak pollen season.

The patient's brain MRI revealed mild to moderate paranasal sinus disease and a small left mastoid effusion; otherwise, the study was unremarkable. Medical records also noted right-sided asymmetrical sensorineural hearing loss.

**Figure 1:**
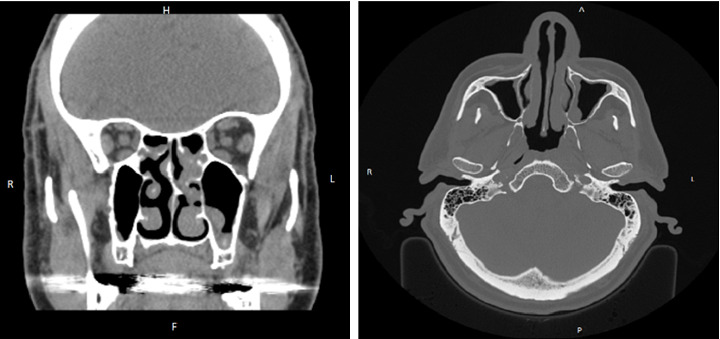
CT sinus study showed moderate to severe paranasal sinus disease, worst at the frontoethmoidal recesses and anterior ethmoid air cells. Leftward nasal septal deviation with bony spurring that contacts the left middle turbinate.

His medical history includes a diagnosis of CRS with nasal polyposis, as assessed by otolaryngology. A CT scan supported the clinical findings, showing a left septal spur, right-sided polypoid changes involving the ostiomeatal complex, bilateral chronic ethmoid sinus fullness, and bilateral obstruction of the ostiomeatal complexes. Additional findings included mucosal thickening of the maxillary, sphenoid, and frontal sinuses (Figure 1).

His records indicate that he underwent a bilateral nasal endoscopy with frontal sinusotomy, total ethmoidectomy, and bilateral sphenoid and maxillary endoscopy with mucous membrane removal. Operative findings confirmed the preoperative diagnosis of bilateral ethmoid polypoid tissue.

The patient reported a few weeks of relief following the surgery but later returned to ENT with recurring symptoms. The ENT specialist confirmed regrowth of turbinate tissue and recommended revision surgery. The patient was started on Dupixent, a dual inhibitor of IL-4 and IL-13, used to treat nasal polyps. A sinus culture was also obtained, revealing *Staphylococcus aureus* resistant to clindamycin. The patient applied Bactroban ointment intranasally and performed daily saline irrigations with Bactine Max, which reportedly provided symptom relief.

After being advised to undergo surgical revision, the patient sought a second opinion, during which a rigid nasal endoscopy was performed. Findings included mucosal edema of the middle turbinate and sinus mucosa; however, no polyps or purulence were noted. Thin mucus stranding was observed along the bilateral inferior turbinates, along with bilateral inferior turbinate hypertrophy. The patient was prescribed doxycycline 100 mg twice daily for 30 days.

At the time of the physical therapy visit, the patient was on his third round of antibiotics since the initial surgery. Historically, he experienced symptom relief with Cosentyx and doxycycline but reported no improvement with previously prescribed steroids. He used Navage nasal irrigation daily. Once a week, the patient underwent hyperbaric oxygen treatment but did not notice any improvement and reported experiencing severe vertigo while in the chamber.

### 2.2. Physical Therapy Initial Examination

Clinical examination demonstrated full cervical range of motion in all planes except for right-side bending, which was limited to approximately 80% of full range. The patient reported soreness in the upper cervical spine during this movement. Passive accessory testing of the right upper cervical spine (C0-2) elicited concordant sinus pain along with a hyperalgesic response to Grade I/II joint mobilizations.

Vestibular examination revealed a positive head impulse test on the right side. Infrared video-oculography detected a third-degree left-beating nystagmus. No significant abnormalities were noted in horizontal or vertical saccades, smooth pursuits, vestibular-ocular reflex (VOR) cancellation, cover/uncover test, or alternate cover test. Positional tests, including Dix–Hallpike, roll test, and cervical torsion test—were all negative.  Table 1 summarizes the results of the oculomotor and vestibular examinations.

Based on these findings, the patient may have right unilateral vestibular hypofunction (UVH), with chronic sinusitis symptoms considered in the differential diagnosis.

### 2.3. Physical Therapy Intervention

The patient was provided with gaze stabilization exercises (VOR x1) to be performed three times daily for a total of 20 min, along with cervical stretches targeting the levator scapulae, upper trapezius, and sternocleidomastoid muscles, performed three times daily with 10 repetitions each, as part of his home exercise program. Due to out-of-town travel, the patient's follow-up treatment was scheduled for 4 weeks after the initial evaluation.

## 3. Outcomes

At 4-week follow-up treatment, the patient had finished doxycycline while also consistent with taking both Cosentyx and Dupixent. Patient denied having any dizziness or vertigo-like symptoms in weeks.

Due to travel, the patient reports inconsistent participation in the exercises provided at the initial evaluation. The vestibular exam demonstrated both a negative head impulse test and negative spontaneous nystagmus.

## 4. Discussion

The patient presented with acute exacerbations of dizziness accompanied by aural symptoms and nausea, without a consistent probable cause. This complex presentation necessitated a thorough evaluation to identify the underlying etiology. The oculomotor examination revealed a diagnosis of right UVH, supported by a positive right head impulse test and third-degree spontaneous nystagmus observed with infrared video-oculography.

Sinus-induced dizziness is a less commonly discussed condition in which inflammation or infection of the sinus cavities may influence the vestibular system, resulting in nystagmus and associated vestibular symptoms. Research suggests that vestibular neuritis may be associated with sinusitis, as pathogens and inflammation in the sinuses could potentially migrate to inner ear through the eustachian tube [13, 14]. Understanding the link between sinus issues and vestibular symptoms is crucial for effective differential diagnosis and treatment. Described in a case study by Haid, 14 out of 15 patients with sinusitis-induced dizziness experienced relief of their dizziness after sinus procedure [8]. These findings suggest a possible link between the sinus condition and vestibular disturbances.

Sinus problems may be found in a thorough medical history review or during physical therapy assessment, allowing physical therapists to identify cases that require referral to an ENT specialist. The evaluation of a patient's sinus history and medications provides valuable discernment on the appropriateness of physical therapy intervention for the management of vestibular symptoms. Physical therapy examination should consist of a detailed patient history, investigating sinus history and its association between vestibular symptoms and sinus implications. Review of current medications can provide insight on chemical compounds and their management of sinus and vestibular symptoms and/or possible side effects.

Surgical intervention may be beneficial for treating anatomical concerns that coincide with chronic sinusitis. In a study by Brody-Camp et al., sinus surgery was performed on 39 subjects (55.7%), with abnormal VNG results in 21 (53.8%) of them. This study demonstrated no significant differences in VNG findings between those who underwent sinus surgery and those who did not [11]. Conversely, patients who underwent surgery experienced a shorter median duration of dizziness symptoms compared to those who did not have surgery (2 months vs. 10 months). Effective management of sinusitis, whether through medical or surgical interventions, can lead to significant improvements in dizziness symptoms.

Clinical physical therapy management of vestibular hypofunction includes gaze stabilization exercises [15]. These exercises aim to improve the VOR and enhance overall stability and gaze control. In cases of no improvement of dizziness symptoms during physical therapy intervention, a physical therapist should consider if there are ongoing pathological conditions that may contribute to the individual's dizziness. Although the patient initially did not participate in the prescribed exercises, the resolution of symptoms following sinus surgery and medication indicates that addressing the underlying sinus pathology was essential.

The limitation of this case report was no vestibular laboratory testing, such as caloric test or VEMP to confirm peripheral vestibular pathologies. Moreover, the patient only had two visits to physical therapy. However, the physical therapies in this case played a consultant role to help with the PCP in identifying possible pathological conditions that required further treatment.

## Figures and Tables

**Table 1:** The results of oculomotor and vestibular examinations.

Oculomotor	Results	Note
Saccade	(−)	
Smooth pursuit	(−)	
Slow VOR	(−)	
VOR cancellation	(−)	No dizziness
Cover/uncover	(−)	
Alternative cover	(−)	
Vestibular		
Spontaneous nystagmus	(+)	Infrared video-oculography revealed a third-degree left-beating nystagmus when looking to the left, without a change in direction.
Head Thrust Test	(+)	To the right
Dix–Hallpike	(−)	
Roll test	(−)	
Cervical torsion test	(−)	
